# Seal of transparency heritage in the CISMeF quality-controlled health gateway

**DOI:** 10.1186/1472-6947-4-15

**Published:** 2004-09-14

**Authors:** SJ Darmoni, B Dahamna, Thomas R Roth-Berghofer

**Affiliations:** 1 CISMeF, Rouen University Hospital, France & L@STICS, PSI Lab FRE CNRS 2645, France CISMeF & L@STICS, 1 rue de Germont 76031 Rouen Cedex, France; 2 University of Kaiserslautern Knowledge-Based Systems Group, Postfach 3049, 67653 Kaiserslautern, Germany

## Abstract

**Background:**

It is an absolute necessity to continually assess the quality of health information on the Internet. Quality-controlled subject gateways are Internet services which apply a selected set of targeted measures to support systematic resource discovery.

**Methods:**

The CISMeF health gateway became a contributor to the MedCIRCLE project to evaluate 270 health information providers. The transparency heritage consists of using the evaluation performed on providers that are referenced in the CISMeF catalogue for evaluating the documents they publish, thus passing on the transparency label from the publishers to their documents.

**Results:**

Each site rated in CISMeF has a record in the CISMeF database that generates an RDF into HTML file. The search tool Doc'CISMeF displays information originating from every publisher evaluated with a specific MedCIRCLE button, which is linked to the MedCIRCLE central repository. Starting with 270 websites, this trust heritage has led to 6,480 evaluated resources in CISMeF (49.8% of the 13,012 resources included in CISMeF).

**Conclusion:**

With the MedCIRCLE project and transparency heritage, CISMeF became an explicit third party.

## Background

The availability of Internet health tools and services has been increasing at a phenomenal rate in recent years making the Internet a major source of knowledge for healthcare professionals, medical students and also patients and the general public. This increase has made it an absolute necessity to continually assess the quality of health information on the Internet. Indeed, creating a Web site is relatively easy, therefore uncontrolled health information can be launched by virtually anyone with access to the Internet. Peer review is often absent throughout this media as opposed to scientific journals. There have been numerous debates about the variable quality of health information on the Internet and its impact on public health [1]. There is no other field in which inaccurate, incomplete, or biased information can be potentially more damaging [2].

In the past five years, a lot of authors have scrutinized the quality of the health content available on the Internet. These studies, assessing the quality of health information, have been extensively reviewed by Eysenbach et al. [1]. In the meantime, several worldwide initiatives have been undertaken to define criteria for assessing the quality of health information on the Internet. These initiatives have been reviewed by Risk and Dzenowagis [3]. However, no consensus has been reached by healthcare professionals or consumers on how to assess the quality of health information on the Internet.

As of today, access to accurate and trustworthy health information on the Internet is not an easy task; there are a great number of directories and search engines available in this new media [4]. But, there is also a need to develop reliable and quality-controlled health subject gateways to disseminate relevant trustworthy health information. Koch [5] defined quality-controlled subject gateways as Internet services which apply a rich set of quality measures to support systematic resource discovery. Considerable manual effort is used to process a selection of resources, which meet quality criteria, and to display a rich description and indexing of these resources with standards-based metadata. Regular checking and updating ensure optimal collection management. The main goal is to provide a high-quality of subject access through resource indexing using controlled vocabularies and via a practical classification structure for advanced searching and browsing.

The objective of CISMeF (French acronym for Catalogue and Index of health resources in French) [6,7] is to describe and index the main health resources in French in order to assist health professionals, students and consumers in their search for electronic information available on the Internet.

CISMeF is a quality-controlled subject gateway initiated by the Rouen University Hospital (RUH). Each of the following phases proposed by Koch [5], which characterise a typical quality-controlled subject gateway, are implemented in CISMeF: (a) selection and collection development, based on the Net Scoring, a list of 49 criteria to assess quality of health information (URL: ) [4], (b) collection management, (c) creation of metadata (performed by experts), (d) resource description (an extensive and documented metadata set), and (e) resource indexing (using a controlled vocabulary system).

CISMeF is manually maintained. In CISMeF, a resource is defined as 1) a Web site or 2) high-quality documents from this Web site. CISMeF describes and indexes the most important sources of institutional health information in French, in order to allow them to be searched quickly and precisely. A great variety of resources are indexed, in terms of resource type (clinical guidelines, teaching material, patients information, etc.), and resource format (html, pdf, etc.). Its Universal Resource Locator (URL) is  The CISMeF Web site opened in February 1995. In December 2003, 13,012 resources had been indexed, with an average of 55 new resources indexed each week. CISMeF is considered by most professionals as the reference health institutional Web site in France with as many as 25,000 unique machines visiting this Web site by working day. Doc'CISMeF is the search tool of the CISMeF gateway [7].

In 1997, because the quality of health information became an important issue for the building and maintenance of a trustworthy health gateway, the CISMeF team participated in the development of a user guide named Net Scoring [8]. The goal of the Net Scoring project was to provide a set of criteria that can be consistently used to assess the quality of health information on the Internet. Between 1997 – 2002 the CISMeF gateway selected health resources using the main criteria established by the Net Scoring initiative (source of information, disclosure, editorial review process, date of last update, and feedback mechanism) in view of the fact that the selection process is mandatory to create a trustworthy health Internet directory. Resources that are not compliant with basic ethical criteria are not included in the CISMeF database.

In 2002, CISMeF became a contributor to the MedCIRCLE project (URL: ) [9]. The aim of this project is to establish a global Web of transparency for networked health information and to increase the accessibility and findability of trustworthy health websites using "Semantic Web" approaches, which essentially means to make "narrative" information on the Web accessible in a machine processable format by using RDF (Resource Description Framework) expressed in XML (eXtended Markup Language) [10]. MedCIRCLE is a collaboration of trustworthy European health subject gateways, medical associations, accreditation, certification, or rating services, which share the common goal of evaluating, describing, or indexing health information. MedCIRCLE began in March 2002 and lasted till December 2003. Whereas CISMeF initially addressed quality on a more finely grained level, i.e., the quality of documents or single web pages, MedCIRCLE focused on whole websites including information about the respective publisher.

The main deliverable of this project for the CISMeF team was the evaluation and rating of 270 health information providers (or publishers) which who release health resources in French on a regular basis. Because CISMeF catalogues and indexes not only Web sites but also and mainly quality-controlled documents from those health publishers, we introduced the concept of "transparency transitivity" or " transparency heritage". It consists in applying to these documents the evaluation performed for their publishers, thus passing on the transparency label from the publishers to their documents.

## Methods

### Providing transparency related metadata

Health professionals have begun to realize that it is their responsibility to guide consumers and patients to the best available medical information on the web. Many national governments and medical societies have acknowledged that it is their responsibility to help users to identify "good quality" information sources and have begun to develop national health gateways (such as HealthinSite in Australia, NHS Direct in the UK, or Healthfinder in the USA), portal sites and other forms of "infomediaries" such as seals of approval [2] or certification mechanisms in an effort to help consumers to locate trustworthy information resources.

However, current approaches do not harness any of the advantages of the Web as a decentralized, distributed information system. There is a need for "next generation" tools, including intelligent knowledge-based tools, allowing consumers to positively and actively identify reliable health information that suits their needs.

The three application partners of MedCIRCLE, besides CISMeF in France, were the Agency for Quality in Medicine (AQuMed) in Germany and the Official Medical College of Barcelona (COMB). AQuMed was founded in March 1995 as a joint institution of the German Medical Association and the National Association of Statutory Health Insurance Physicians. AQuMed established a health gateway (URL: ) for laypersons, listing consumer health information sites. Before MedCIRCLE, documents had been evaluated using the DISCERN instrument [11]. COMB (URL: ) represents the medical profession of Barcelona. To this date, in the project "Web Medica Acreditada", COMB has accredited more than 300 Spanish health websites from Spain and Latin America [12]. The Knowledge Management Department of the German Research Center for Artificial Intelligence DFKI GmbH provided consultancy services especially in the area of ontology modeling. DFKI also provided the technical infrastructure and development resources for the project.

### CISMeF terminology

The CISMeF team is composed of five medical librarians, two medical informaticians, one engineer, three Ph.D. and two Master students in Computer Science. CISMeF uses two standard tools for organizing information: the MeSH (Medical Subject Headings) thesaurus from the US National Library of Medicine, and several metadata element sets [13]: (a) 11 of 15 items of the Dublin Core metadata format to describe and index all the health resources included in CISMeF (author or creator, date, description, format, identifier, language, publisher, resource type, rights, subject and keywords, and title), (b) the 11 elements of the Educational category from Learning Object Metadata (LOM) for teaching resources, (c) specific metadata for evidence-based medicine resources (indication of the level of evidence and the method to calculate it) which also describe the health content [14], and (d) the HIDDEL metadata set (Health Information Disclosure, Description and Evaluation Language) [15].

### Description of the HIDDEL language

HIDDEL is a metadata language and an ontology, which enables the expression of descriptive and evaluative annotations in XML/RDF. The first version of HIDDEL was initially developed during the MedCERTAIN project (MedPICS Certification and Rating of Trustworthy Health Information on the Net, ) [16]. HIDDEL evolved from MedPICS [17], a basic rating vocabulary for medical information conforming to the Platform for Internet Content Selection (PICS) [18]. HIDDEL is used to enhance transparency of health information on the Internet.

HIDDEL is based on existing quality criteria such as the Health On the Net (HON) Code of Conduct [2]. It was developed together with a quality management process model. HIDDEL can be used by information providers for self-disclosure, but also by third parties such as quality-controlled health gateways, to *evaluate *health information providers. It presents three levels of evaluation: (a) self-disclosure (b) evaluation by non-medical experts, and (c) evaluation by medical experts. As a quality-controlled subject gateway, CISMeF uses HIDDEL only as a third-party.

The HIDDEL vocabulary can be downloaded freely from the MedCIRCLE Web site, as long as the sources are acknowledged and requests for changes or expansions are fed back to the community. At present HIDDEL is available in four languages: English, German, French and Spanish. The use of this controlled vocabulary enables automatic translation (except for free text). The heritage process was made possible because of HIDDEL's dual structure: on the one hand, Infoprovider metadata, describing to the health information provider (e.g., the name of the person responsible for the quality of the web site), and on the other hand, Sitespecific metadata devoted to one Web site evaluation (e.g., language). In CISMeF, we have applied Sitespecific metadata to each resource (mainly quality-controlled documents) from a publisher already included in the CISMeF database. The name of the person responsible for the quality of the Web site, which is one of the Infoprovider metadata, is the same for every document of the Web site. On the contrary, the language of the document, which is one of the Sitespecific metadata, may vary from one document to another. The CISMeF team implemented the whole HIDDEL structure in the CISMeF database, which involved the creation of triggers, thus ensuring automated transfer from CISMeF to HIDDEL metadata, and the creation of new forms (interface recasting) to deal with non-CISMeF metadata. Because the HIDDEL elements are optional and repeatable, CISMeF has selected a number of 70 metadata among the 305. Most of the metadata previously used in CISMeF and in particular the Dublin Core are also included in the HIDDEL language. These metadata were automatically triggered in the HIDDEL language.

### Interoperability

The interoperability process consists of an exchange of RDF files, containing experts' annotations "written" in HIDDEL. The semantic-based Archer Annotation System deals with RDF annotations reception. Archer is a Web application that allows annotating health information Web sites using the HIDDEL vocabulary. It is a technical platform and an organizational infrastructure that can be used by consumers, health information providers, and third party rating services. The first version of Archer was implemented as a part of MedCERTAIN, and further enhanced in the course of the successor project MedCIRCLE to allow the exchange of metadata between third party rating organizations.

**Figure 1:**
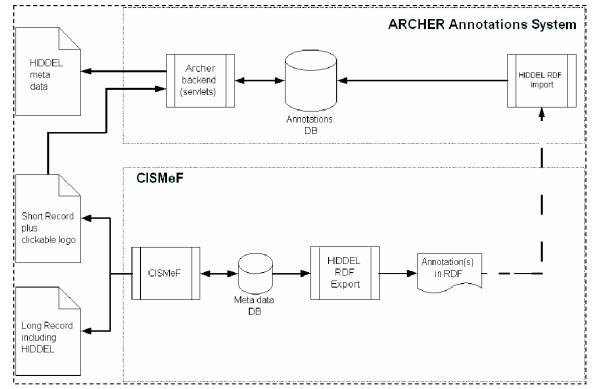
Interoperability between CISMeF and Archer

On another ground, through its search engine Doc'CISMeF, CISMeF provides external links to Archer backend servlets, and internal links to rated sites disclosure (see Figure 1). Since August 2002 the CISMeF team has embedded RDF metadata (URL: ) into the generated HTML pages, making them not only machine-readable (as every HTML page is) but also machine-processable. Therefore, one of the main goals of this metadata element set was fulfilled easily: it became interoperable with other Internet services. Moreover, an RDF Scheme describing CISMeF specific metadata was created (URL: ).

**Figure 2:**
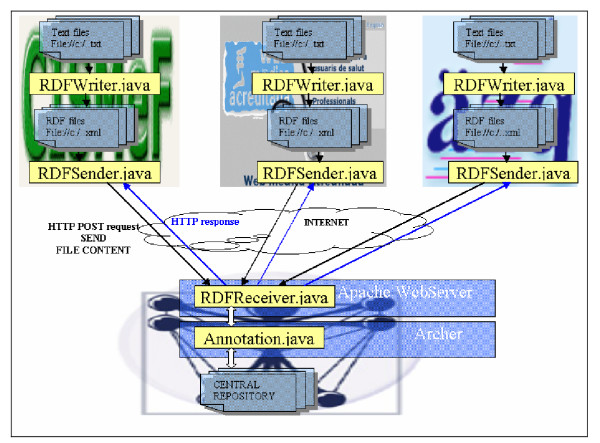
Interacting java classes to export rating results into the central repository

In a more pragmatic way, interoperability relies on a 3 steps process (see Figure 2): (1) RDF files generation: a Java program (RDFWriter.class) formats evaluation data according to a MedCIRCLE RDF Schema of annotations; (2) RDF files export: a Java program (RDFSender) sends RDF files to the MedCIRCLE web server using HTTP Post; (3) Reception and ID allocation: for each transmitted file, the MedCIRCLE Web server sends back an ID number that will be used to access the exported metadata.

## Results

The CISMeF team in the MedCIRCLE consortium has evaluated and annotated the main health information providers (or publishers) included in the CISMeF database: national agencies, medical societies, universities and hospitals. CISMeF first checks the publishers' information without asking the health information provider to self-declare any metadata as described in the MedCERTAIN quality management process.

CISMeF used HIDDEL to select and evaluate the 270 health publishers most represented in CISMeF and made the results of their evaluations explicit and accessible using RDF metadata. These were exported into the searchable MedCIRCLE Open Directory.

**Figure 3:**
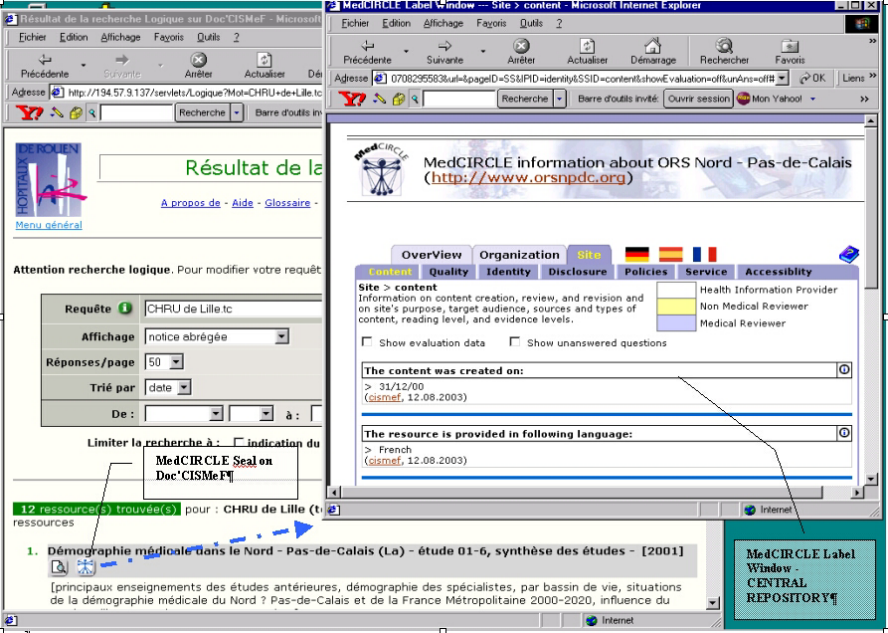
Links between Doc'CISMeF and MedCIRCLE repository

Each site rated in CISMeF has a record in the CISMeF database that generates an RDF into HTML file. The search tool Doc'CISMeF (URL: ) displays the information originating from each of the publishers that were evaluated with a specific MedCIRCLE button, which is linked to the MedCIRCLE central repository where HIDDEL metadata elements are displayed (see Figure 3).

Seal of trust, such as the one developed by HON, is a "quality seal" or a "seal of approval": i.e. the HON logo provides an accreditation, whereas the MedCIRCLE seal is not a "quality seal" but a "transparency seal": it is a button allowing health professionals and consumers to access metainformation. The presence of a MedCIRCLE button on a health Web site does not imply, in any way, that the site meets minimum required standards. This decision is left up to the user. In contrast, a seal of trust is a quality seal: i.e. every Web site with a seal of approval (such as the HON seal) has been previously accredited a third party.

Nonetheless, every resource included in CISMeF and those evaluated by the MedCIRCLE process are quality-controlled. The MedCIRCLE consortium takes a very neutral approach and does not impose but strongly recommends certain procedures or minimum metadata, taking into account that collaborating gateways, accreditors, certifiers, raters may approach from very different angles.

CISMeF has applied full heritage from the evaluated publishers: each document from a MedCIRCLE rated publisher, indexed in CISMeF, will also receive the MedCIRCLE button of the publisher with the same link to MedCIRCLE central repository. The idea is to keep the common Infoprovider elements for every document, and to use CISMeF metadata to disclose Sitespecific elements specific to each document, in particular the indexing with the MeSH thesaurus. At the end of the project in December 2003, starting from 270 websites, the translation from CISMeF metadata to HIDDEL, led to 6,480 evaluated resources in CISMeF in September 2003 (49.8% of the 13,012 resources included in CISMeF). All CISMeF selected HIDDEL metadata (70 out of 305) are displayed in the Doc'CISMeF record in RDF into HTML. The top five publishers indexed in CISMeF, which produced trustworthy documents in French are: Grenoble Medical School (N = 435), Health Canada (n = 275), Strasbourg Medical School (N = 263), and French Ministry of Health (N = 248).

Every new document (e.g. clinical guideline or teaching material) included in CISMeF from one of the 270 main publishers evaluated through the MedCIRCLE process inherits automatically: (a) a MedCIRCLE button linking to the repository and (b) HIDDEL metadata included in CISMeF database and displayed in the Doc'CISMeF record in RDF into HTML. On the other hand, a document that comes from one of the 270 publishers evaluated within the MedCIRCLE project but not included in the CISMeF database will not receive a MedCIRCLE button in the CISMeF gateway. However, this more global transitivity could be applied in a more generic search tool such as Google.

Since February 2003, when the MedCIRCLE button became operational in the CISMeF gateway, the CISMeF team has decided to go on (keep) applying the MedCIRCLE transparency process after the end of the project with the following rules: (a) check every month if there is a new publisher with five documents already included in CISMeF and (b) if so, begin the MedCIRCLE process for these publishers and apply transparency heritage for their respective documents. We applied these rules after the end of the EU-funding project. In March 2004, the CISMeF database contained a complete evaluation through the MedCIRCLE process for 346 publishers (+76, as compared to the EU-grant proposal) and 7,053 documents from these publishers (53.3% of the 13,227 resources included in CISMeF).

In the MedCIRCLE repository, end-users can access an aggregate view of what people say about a certain Web site by clicking: The CISMeF gateway is one of many possible producers of trustworthy metadata regarding a health information provider. Metadata from the Open Directory can also be fed into search engines and other gateways.

## Discussion

As the number of health related Web sites worldwide has been estimated as being around 100,000, complete coverage by a single third party evaluation body is impossible. Instead, a collaborative approach as shown in this paper has to be promoted, whereby different rating services or organizations use comparable standards and a common metadata language. More recently, the Health on the Net Foundation has developed a HON tool bar in the course of the EU-funded Active Health (Active Environment for Health Promotion and Disease Prevention, URL: ) consortium (URL: ): this HON tool bar is indicating if the site is accredited directly by HON or indirectly by one specific accredited Web sites as health gateways such as CISMeF or MedlinePlus. In this context, HON is creating a seal of quality trust heritage where MedCIRCLE is creating a seal of transparency heritage. A formal evaluation of these two examples of heritage (quality seal vs. transparency seal) is mandatory to check their respective hypothetical added value.

One of the main findings of the MedCIRCLE consortium in the course of the project has been that there is no absolute objective quality of a Web site. Quality is to a certain degree subjective, may vary in time and also according to the eye of the beholder. A Web site that a consumer looking for health information finds acceptable one day may be unacceptable another day. For example, a consumer may search general information for one drug (e.g. after a TV show) and finds advice on a patient information Web site. Later, the same consumer searches for the same drug for his/her child. But this time, he/she checks if the respective Web site is sponsored by a pharmaceutical company. He/she comes to the same Web site as before, but this time this Web site will be unacceptable. The context of the search changed, making a general advice acceptable and a specific advice unacceptable. By providing metadata about health related websites, MedCIRCLE allows the health information consumers to decide themselves if a website is of good quality. Trust is improved by enhancing transparency. More globally, the MedCIRCLE consortium will lead to a safer Internet by providing a seal of transparency to over 7,000 resources on the Internet via the CISMeF search engine. The precaution principle is now widely accepted in the European Union, which develops Action Plans, such as the Action Plan for Safer Use of the Internet (URL: ), which partially granted the MedCIRCLE consortium.

The MedCIRCLE button does not directly fulfill the objective to only identify reliable health information. This objective is in fact fulfilled by th HON initiative. In contrast, the MedCIRCLE consortium indirectly fulfill this objective (i.e. to identify only reliable health information) by providing a seal of transparency. The Netizen (citizen on the Internet) has the active role to evaluate the reliability when reading the HIDDEL metadata after clicking the MedCIRCLE button. With a seal of trust, the role of the Netizen is more passive but leads to a faster trust information access. Nevertheless, a drawback of the MedCIRCLE button is a possible misunderstanding by end-users, who might confuse it with a quality seal and therefore may forget to actively press the MedCIRCLE button, which may hinder their valid judgement. Here again, a formal evaluation of theses two approaches (transparency vs. trust) is necessary.

While using Net Scoring, CISMeF was acting as an *implicit *third party. One of the main results of the MedCIRCLE project, from the CISMeF team's point of view, is that CISMeF proceeded from being an implicit to being an *explicit *third party thanks to the creation of the MedCIRCLE button now used for 346 publishers (2.6% of the resources included in CISMeF) and 7,053 resources (53.3% of the resources included in CISMeF), which multiplies by 20 the number of resources with a seal of trust.

The HIDDEL language is totally embedded in the CISMeF metadata element sets. This allows a very easy interoperability with MedCIRCLE tools, and more specifically Archer. Semantic Web approaches already used in the MedCIRCLE project could open up new ways for educating health professionals and consumers and reaching less savvy health professionals and consumers, because part of the intelligence and knowledge currently required to critically appraise information on the health professional or consumer Web site could be built into the search tools. The feasibility of this approach has been already demonstrated by CISMeF but also by the German (AQuMed) and Spanish partners (COMB). The impact on health professionals and consumers is subject to ongoing investigation within the MedCIRCLE project. The Semantic Web may provide the health professional or the consumer with a greater capacity to determine the reliability of a given health information provider or service than the Web in its current form.

## Conclusion

With the MedCIRCLE project and transparency heritage, CISMeF became an explicit third party.

## Competing interests

None declared.

## Authors' contributions

SJD had the original idea of seal transparency and drafted the manuscript. DH and TRRB developed respectively in France and Germany the programs to implement the seal transparency in CISMeF & MedCIRCLE Web sites.

## Pre-publication history

The pre-publication history for this paper can be accessed here:
